# Role of neutrophils in the pathogenesis of Post Kala-azar Dermal Leishmaniasis (PKDL)

**DOI:** 10.1371/journal.pntd.0012655

**Published:** 2024-11-27

**Authors:** Madhurima Roy, Ritika Sengupta, Bidhan Chandra Chakraborty, Uttara Chatterjee, Esther von Stebut, Paul M. Kaye, Mitali Chatterjee

**Affiliations:** 1 Dept. of Pharmacology, Institute of Post Graduate Medical Education and Research (IPGME&R), Kolkata, India; 2 Multidisciplinary Research Unit (MRU) Institute of Post Graduate Medical Education and Research (IPGME&R), Kolkata, India; 3 Pathology, Institute of Post Graduate Medical Education and Research (IPGME&R), Kolkata, India; 4 Department of Dermatology, Medical Faculty, University of Cologne, Cologne, Germany; 5 York Biomedical Research Institute, Hull York Medical School, University of York, York, United Kingdom; Aarupadai Veedu Medical College & Hospital, INDIA

## Abstract

**Background:**

Post Kala-azar Dermal Leishmaniasis (PKDL) is a dermal sequel of visceral leishmaniasis (VL), poses a significant threat to the success of ongoing kala-azar elimination program, due to its potential role in sustaining transmission cycles and complicating disease management strategies. In VL, neutrophils have been identified as the ‘first line of defence’, having multiple roles in disease pathogenesis, but their role in PKDL, if any, still remains elusive; presenting a critical gap in knowledge, and was the aim of this study.

**Methodology/Principal findings:**

In a cohort of PKDL patients, CD66b^+^ neutrophils were quantified in skin biopsies, followed by immunostaining of FFPE sections to identify activated neutrophils (CD66b^+^/CD64^+^) and degranulated (CD66b^+^/MPO^+^), along with expression of neutrophil elastase (NE), matrix metalloprotease 9 (MMP9) and collagen I. Plasma levels of neutrophil chemo-attractants CXCL8/1/2/5, CCL2 and 20 and cytokines, (IL-6, IFN-γ, IL-4, IL-10, TNF-α, IL-17 and IL-22, 23) were evaluated by a multiplex assay, while lesional expression of IL-8, IL-10 and IL-17 was evaluated by immunohistochemistry. As compared to healthy individuals (control skin samples), PKDL cases at the lesional sites had an increased number of activated CD66b^+^ neutrophils (positive for CD64^+^, MPO^+^ and NE^+^). The plasma levels of neutrophil chemo-attractants, pro-inflammatory and regulatory cytokines were raised as was circulating and lesional IL-8, along with an enhanced lesional expression of IL-10 and IL-17A. An increase in circulatory and lesional MMP9 was accompanied by decreased collagen I, suggesting disintegration of matrix integrity.

**Conclusions/Significance:**

Taken together, in PKDL, activated neutrophils possibly contribute towards modulating the lesional landscape. Understanding this involvement of neutrophils in patients with PKDL, particularly in the absence of an animal model, could offer better understanding of the disease pathogenesis and provide insights into novel therapeutic strategies for the ongoing elimination program.

## Author summary

Visceral leishmaniasis (VL), also known as kala-azar, is a fatal form of leishmaniasis caused by the digenetic parasite *Leishmania donovani*. In South Asia, around 10–20% of the cured VL cases develop a chronic dermal manifestation known as post-kala-azar dermal leishmaniasis (PKDL). Patients with PKDL serve as ‘active disease reservoirs’, perpetuating the transmission cycle and thereby pose a significant threat to the success of the ongoing kala-azar elimination program. Moreover, the absence of an animal model in PKDL poses a great challenge in elucidating the pathogenesis of the disease, thereby restricting studies exclusively to clinical cases. Neutrophils are recognized as crucial players in the initial phase of VL, acting as the ‘first line of defence’ and actively influence the outcome of infection, but their significance in PKDL has been overlooked, with previous research primarily focussing on macrophages and lymphocytes. This study addresses the critical gap in understanding the role of neutrophils in PKDL. This study revealed that neutrophils infiltrating at the lesional sites in PKDL played a functional role in modulating the disease pathology, shedding light on their involvement beyond the initial phase of infection. Conclusively, the findings in this study highlighted the importance of considering neutrophils as the ‘new kid in the block’ in PKDL immunology. In addition, this study contributed to a broader understanding of the disease pathogenesis by elucidating the extended roles of neutrophils in chronic phase of infection providing valuable insights that could potentiate targeted interventions, crucial for elimination of this debilitating neglected tropical disease.

## Introduction

The leishmaniases, caused by the protozoan parasite *Leishmania*, comprise a diverse group of neglected tropical diseases with clinical pleomorphism(s) ranging from self-healing cutaneous leishmaniasis (CL), non-healing mucocutaneous leishmaniasis (MCL), to fatal visceral leishmaniasis (VL). Post kala-azar dermal leishmaniasis (PKDL), caused by *Leishmania donovani*, presents in patients with apparently cured VL and is possibly the most challenging variant, especially in terms of its etiopathogenesis [1–3]. Patients with PKDL present with either papulonodular (polymorphic) or hypomelanotic lesions (macular), that harbour parasites in the skin, which being easily accessible to sandflies, act as potential disease reservoirs and are proposed to play a major role in transmission. This disease is confined to two disjointed geographic zones, South-East Asia and East Africa (mainly Sudan). In South-East Asia, approximately 2.5–20% of apparently cured VL patients develop PKDL several years later, whereas in Sudan, it occurs concomitantly with VL in around 20% [4,5]. Active surveillance in India has reported the prevalence of an equal proportion of polymorphic and macular cases of PKDL, indicating that the latter form can no longer be considered as an uncommon presentation [2]. PKDL’s role as a reservoir poses a major challenge in the ongoing kala-azar elimination programme across South-East Asia that is currently set for 2030 [Global leishmaniasis surveillance: 2019-2020, a baseline for the 2030 roadmap (who.int), accessed on 19^th^ February 2024].

The development of leishmaniasis hinges on the inability of host microbicidal mechanisms to eliminate parasites. Neutrophils are the foremost to arrive at the site of infection after a sandfly bite [6,7], and their potent arsenal during the early phase of infection includes phagocytosis, generation of reactive oxygen species (ROS) and extrusion of neutrophil extracellular traps (NETs) [8]. Conversely, neutrophils may also facilitate infection by serving as conduits for transfer of parasites to macrophages [9,10]. Additionally, *Leishmania donovani* can hijack neutrophils and traffic themselves into non-lytic compartments, enabling their subsequent transfer into macrophages [11]. Although *Leishmania amazonensis* promastigotes can induce NETs by their surface lipophosphoglycan (LPG), and are eliminated by neutrophils [12], *L*. *donovani* conversely utilises 3′-nucleotidase to digest the DNA scaffold and escape NET-mediated killing [12]. However, with reference to *L*. *amazonensis* [10], *L*. *mexicana* [13] and *L*. *infantum* [14], amastigotes demonstrate strong resistance against the microbicidal effects of neutrophils. In addition, neutrophils secrete a wide array of cytokines and chemokines that aid recruitment of adaptive immune cells [15], and can shape adaptive immunity via their ability to regulate T cell proliferation [16], as also enhance B cell immunoglobulin class-switching [17]. These latter aspects of neutrophil biology are less well studied in the context of leishmaniasis. Taken together, depending on the parasite species and life cycle stage, neutrophils can contribute towards disease persistence and/or host protection [18–20].

Clinical studies regarding the immunoregulatory role of neutrophils in VL identified immature, highly activated and degranulated neutrophils with limited effector functions [21]. Additionally, in Indian VL, a subset of low-density circulatory neutrophils was identified that expressed markers of antigen presentation, including MHC Class II molecules, co-stimulatory molecules CD80 and CD86, along with augmented expression of arginase 1 and IL-10 [22]. In chronic CL, the role of neutrophils is well recognized as accompanying IL-17 dependant disease progression [23]. However, in PKDL, the role of neutrophils remains poorly understood, probably owing to an absence of an animal model that can simulate the dermal pathology. Accordingly, this study aimed to assess tissue biopsies from patients with PKDL to characterise the extent of neutrophil infiltration and associated markers related to neutrophil recruitment.

## Materials and methods

### Ethics statement

The study received approval from the Institutional Ethics Committee of Institute of Post Graduate Medical Education & Research (IPGME&R), Kolkata. All experiments were performed in accordance with relevant guidelines and regulations. Written informed consent was obtained from all patients or their legally acceptable representative (if <18years).

### Reagents

All reagents were from Sigma Aldrich (St. Louis, Mo, USA) except anti-human Neutrophil Elastase (NE, clone EP223), anti-human Matrix Metalloproteinase 9 (MMP9, clone EP127, Bio SB, CA, USA), Collagen 1 (Abclonal, MA, USA), secondary detection system CRF Anti-Polyvalent HRP Polymer (DAB) stain kit (ScyTek Laboratories, UT, USA), anti-human CD66b-Fluorescein isothiocyanate (FITC), CD64-Phycoerythrin (PE), MPO-PE (BD Biosciences, CA, USA), MMP9 Human ELISA Kit (RayBiotech, GA, USA), Hoechst/trihydrochloride-trihydrate (Invitrogen, MA, USA), rK39 immunochromatographic strip test (InBios International, WA, USA), QIAmp DNA Mini kit (Qiagen, Hilden, Germany), SYBR Green qPCR Master Mix (Applied Biosystems, NY, USA) and Bio-Plex Pro Human Chemokine Panel 40-Plex (for CXCL8/1/2/5, CCL2, 20, IL-6, IFN-γ, IL-4, IL-10, TNF-α, IL-17, 22 and 23, BioRad, CA, USA).

### Study population

Patients clinically diagnosed with PKDL (n = 24) were recruited from the Dermatology outpatient departments of School of Tropical Medicine (STM), Kolkata (n = 3, 2004 onwards) or during active field surveys (n = 21, 2015 onwards) [24]. The initial diagnosis was empirically based on clinical features, a prior history of VL, rK-39 positivity, and/or if they resided in an area endemic for VL. Subsequently, diagnosis was confirmed by *k-DNA* qPCR in skin biopsies excised from the suspected PKDL cases, as previously described [25]. None of the patients suffered from any identifiable co-infections or inflammatory conditions and pregnant women were excluded.

Dermal biopsies (4 mm) were obtained for (i) isolation of DNA and diagnosis by qPCR and (ii) preparation of a FFPE block for immunohistochemistry/immunofluorescence-based assays. These biopsies were procured from macules for macular PKDL cases, whereas in the polymorphic form, it was usually from a papule or nodule. Overall, all biopsies were acquired from sites manifesting active clinical features of PKDL.

Healthy volunteers (n = 10) were recruited from non-endemic areas, and were negative for anti-leishmanial antibodies. Skin biopsies (n = 7) from foreskin of males undergoing voluntary circumcision were included as healthy controls [26]. Studies have endorsed that the preputial skin histology is similar to adult skin [26–28] and can be used as a healthy counterpart. Usually, patients were agreeable to provide a maximum of two biopsies, one for diagnosis (quantification of parasite burden) and another for examining the histopathology. Therefore, collection of an additional skin biopsy from a non-lesional area was challenging. Owing to limited availability of biological material, all markers could not be evaluated in each patient; samples were randomly selected for determination of individual parameters, ensuring at least 5–6 samples were analysed per assay.

### Immunofluorescence (IF) and Immunohistochemistry (IHC)

Formalin fixed paraffin embedded (FFPE) tissues were initially examined by Hematoxylin and Eosin (H&E) for determining the extent of cellular infiltration [27] and were processed as previously described [28]. For IF, after heat-induced epitope retrieval at pH 6.0 with citrate buffer, the slides were stained overnight at 4°C with fluorochrome conjugated primary antibodies [CD66b-FITC, CD64-PE and Myeloperoxidase-PE (MPO-PE), diluted 1:100] or primary antibody (Collagen I diluted 1:200), followed by incubation with fluorochrome conjugated secondary antibody (IgG-FITC, diluted, 1:200) for 2 hr. Slides were counterstained with Hoechst (1.0 μg/ml); sections from human spleen served as a positive control for CD66b and human tonsil for CD64 and MPO.

For IHC, FFPE sections were processed as previously described [27], incubated with pre-diluted primary antibodies [Neutrophil elastase (NE) and Matrix metalloproteinase 9 (MMP9), 50 μl, 1hr], IL-8 (diluted, 1:300), IL-10 (diluted, 1;100) and IL-17 (diluted, 1:500), and processed as per the manufacturer’s protocol; detection was done using alkaline phosphatase (AP, red chromogen) for IL-8 and IL-17A, while for NE, MMP9 and IL-10, 3,3’-diaminobenzidine (DAB, brown chromogen) was used. For NE and MMP9, sections from human spleen and for IL-8, 10, 17, skin from psoriasis patients served as positive controls.

As the study aimed to assess the degree of inflammation, non-inflammatory tissue from circumcised skin served as the comparator arm, while human spleen or tonsillar tissue was the positive control for studying the infiltration of neutrophils and their functionalities. Other inflammatory diseases e.g., leprosy, eczema, psoriasis are expected to demonstrate similar infiltration patterns. In addition, for assessing the presence of cytokines in tissue, skin from patients with psoriasis served as positive controls.

### Imaging analysis

For histopathology and immunohistochemistry-based assays, imaging was performed using a light microscope (EVOS FL Cell Imaging System, Thermo Fischer Scientific, MA, USA) at 10X and 40X magnification. Quantification in tissues (H&E, IHC, IF) was done by QuPath (Quantitative Pathology), an open-source software as a tool for image analysis [(version 0.4.2, Belfast, Ireland), 29]. The total cellular infiltration was counted wherein five fields were evaluated at 40X, averages obtained and expressed as total number of positive cells/field of view (FOV). For H&E staining, each Hematoxylin positive cell nuclei accounted as a ‘positive cell’, and for IHC/IF, chromogen/fluorescence, positive cells counterstained with Hematoxylin/Hoechst positive nuclei were considered as ‘positive cells’. For cytokines and markers diffusely present in lesional sites, individual cell positivity was not performed; instead, their expression was stated as positive or negative. Similarly, for immunofluorescence analysis using collagen 1, individual cell analysis was not feasible as staining was diffuse, and was expressed as mean fluorescence intensity, measured in terms of positive fluorescence /field at 40X magnification as measured by QuPath. All analysis using the QuPath software were performed using the ‘merged’ FITC-PE images, and a constant sigma factor as also threshold intensity cut-off was maintained. The brightness and contrast settings applied to ‘healthy control’ images were identical for ‘PKDL’ images. Imaging for immunofluorescence assays was by confocal microscopy (TCS SPE, Leica Microsystems, WZ, Germany) or THUNDER imaging systems (Leica Microsystems, WZ, Germany) at 10X/20X and 40X magnification respectively. To minimize bias, two blinded investigators independently evaluated the slides.

### Quantification of circulating cytokines and chemokines

Plasma levels of IL-8/CXCL8, 1, 2, 5, CCL2, 20, IFN-γ, IL-6, 4, 10, 17, 22, 23 and TNF-α were measured using a multiplex detection kit (samples diluted in the ratio of 1:4 in sample diluent) as per the manufacturer’s protocol. Data was acquired in a Luminex 200 Labmap system (Luminex, TX, USA) and analysed using BioPlex Manager software version 6.2; the cytokine concentrations were interpolated from an appropriate standard curve. In all analyses, an internal control was incorporated to evaluate reproducibility. Circulating levels of MMP9 were measured using a commercially available kit (RayBiotech, GA, USA) as per manufacturer’s protocol. Absorbances were measured at 450 nm using an ELISA reader (Merilyzer EIAQuant, Meril Life Sciences, India); sensitivity of detection was 74.07 pg/ml.

### Measurement of parasite load by real time PCR (qPCR)

For measurement of parasite load, a standard curve was generated by adding a defined number of *Leishmania* parasites sourced from a *L*. *donovani* strain (MHOM/IN/83/AG83), ranging from 10 to 1 × 10^5^) to blood (180 μl) sourced from a healthy control. Real-time PCR was performed using specific primers for minicircle kDNA (116bp, forward 5’- CCTATTTTACACCAACCCCCAGT-3’and reverse 5’-GGGTAGGGGCGTTCTGCGAAA-3’) as previously described [25].

### Statistical analysis

Results were expressed as median (Interquartile range, IQR) rounded to the nearest integer and non-parametric data was analysed between groups by Kruskal Wallis test followed by Dunn’s multiple comparison test; while for parametric data, ordinary one-way ANOVA followed by Tukey’s Multiple Comparison Test was performed. For analysis of two groups, data were analysed using Student’s t-test (unpaired t-test) for parametric data and Mann Whitney test for non-parametric data. Correlation was assessed by Pearson’s or Spearman’s rank correlation for parametric and non-parametric data respectively; a coefficient correlation of r ≥0.4 was considered relevant. All statistical analysis was performed using GraphPad Prism software (version 8.4.2, GraphPad software Inc., La Jolla, CA, USA), p<0.05 being statistically significant.

## Results

### Study population

The study population (n = 24) included a comparable proportion of patients with polymorphic (n = 12) and macular PKDL (n = 12), along with age and sex-matched healthy controls (n = 17) to mimic the clinical scenario [2,30]. All cases reported a prior history of VL, and a median lag period i.e., gap between manifestation of PKDL lesions and completion of treatment for VL of 3.00 and 2.50 years, for macular and polymorphic PKDL respectively (Table 1). The median disease duration for both forms of PKDL was 2.00 years, and the parasite load in the polymorphic form was higher than macular PKDL (p<0.01, Table 1). PKDL cases (13/24) received sodium antimony gluconate (SAG) during treatment for VL and were recruited between 2006–2017. Due to development of widespread resistance to SAG, World Health Organization (WHO) expert committee on Leishmaniasis and the Regional Technical Advisory Group (RTAG) for the WHO South-East Asia Region (SEAR) subsequently recommended use of Liposomal Amphotericin B (LAmB) as the first-line treatment for kala-azar in the Indian Sub-continent, with a single 10 mg/kg dose (https://ncvbdc.mohfw.gov.in/WriteReadData/l892s/opertional-guideline-KA-2015.pdf, accessed on 19th August, 2024).

**Table 1:** Study population.

Clinical features	Patients with PKDL [n = 24]		Healthy controls [n = 17]
	Macular PKDL [n = 12]	Polymorphic PKDL [n = 12]	
Age, years*	23.50 (14.00–30.50)	27.50 (19.25–39.75)	25.50 (3.10–30.50)
Sex (Male: Female)	7:5	8:4	9:8
History of VL	12/12 = 100%	12/12 = 100%	NA
Treatment during VL	SAG: 7/12 LAmB: 3/12 Unable to provide information: 2/12	SAG: 6/12 LAmB: 4/12 Unable to provide information: 2/12	NA
Lag period, years*	3.00 (2.00–7.50)	2.50 (1.00–18.00)	NA
Disease duration, years*	2.00 (1.00–5.00)	2.00 (1.00–4.00)	NA
Parasite load (parasites/μg genomic DNA)*	7653^#^ (2423–150046) (10/12)	10575^#^ (2213–474863) (10/12)	NA

Skin biopsies were collected from suspected cases of PKDL as described in materials and methods.

*Values are stated as median (IQR); Lag period is the interval between cure from VL and onset of features of PKDL; Disease duration is the time between the onset of PKDL and inclusion in this study.

^#^Significance p<0.01 between parasite load of macular and polymorphic PKDL, VL: Visceral Leishmaniasis PKDL: Post kala-azar dermal leishmaniasis, SAG: sodium antimony gluconate, LAmB: liposomal amphotericin B, NA: not applicable.

### Status of cellular infiltrate

**Fig 1:**
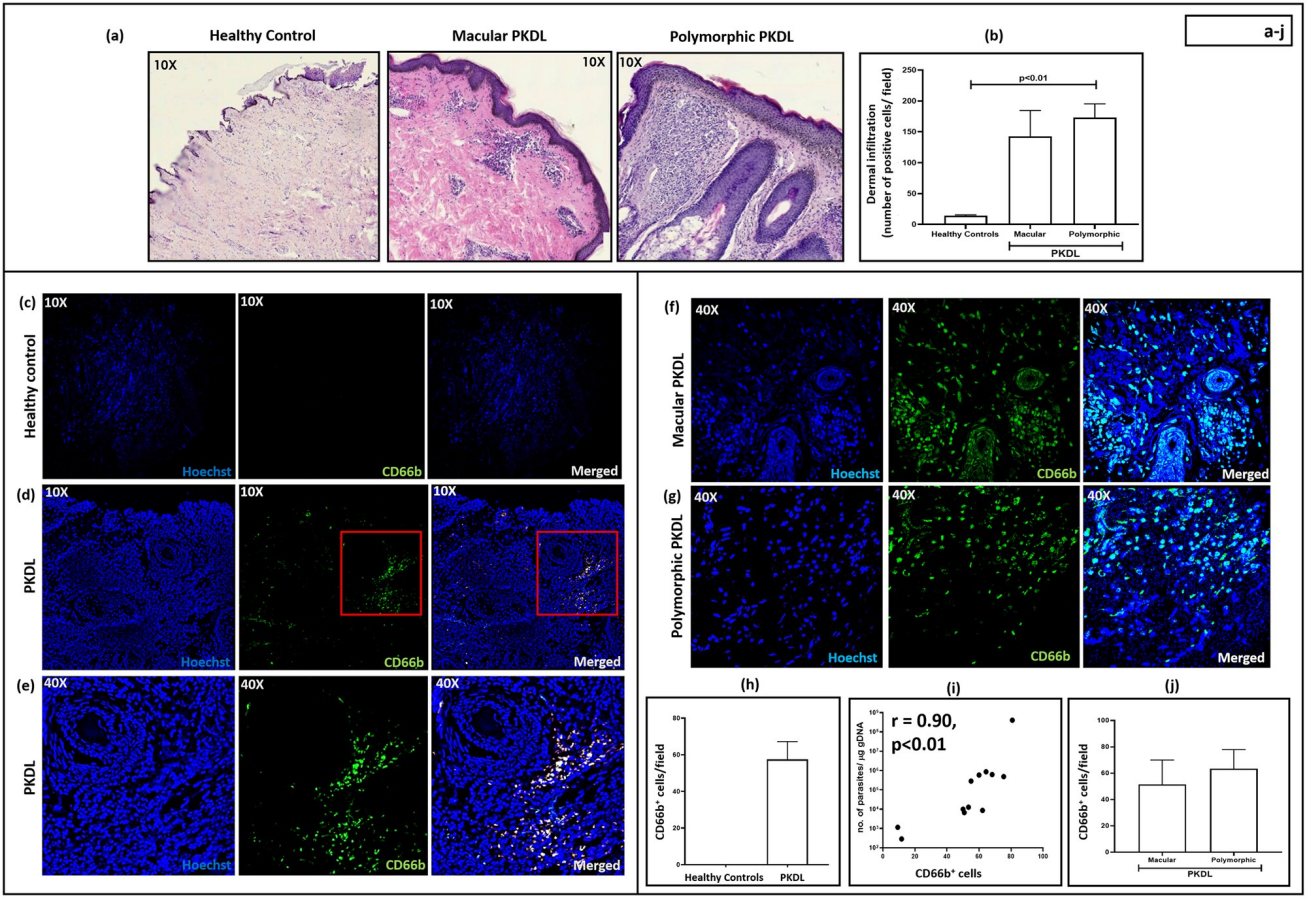
Status of lesional CD66b^+^ neutrophils in patients with Post Kala-azar Dermal Leishmaniasis (PKDL). **(a):** Representative profiles of H&E stained sections from dermal biopsies of a healthy individual (n = 6), patient with macular (n = 6) and polymorphic PKDL (n = 6, 10X magnification). **(b)** Bar graphs indicating the total dermal infiltration in healthy controls (n = 6), macular (n = 6) or polymorphic (n = 6) PKDL. Each horizontal bar represents the median (IQR). **(c):** Representative immunofluorescence profiles of CD66b^+^ cells from dermal biopsies of a healthy individual (10X magnification), stained with CD66b (green) and Hoechst (blue). **(d-e):** Representative immunofluorescence profiles of CD66b^+^ cells from dermal biopsies of a patient with PKDL at 10X (**d**) and 40X magnification (**e**), stained with CD66b (green) and Hoechst (blue). The red squares in 10X images **(d)** represent the area that was imaged at 40X magnification **(e)**. **(f-g):** Representative immunofluorescence profiles of CD66b^+^ cells from dermal biopsies of a patient with macular **(f**) and polymorphic PKDL (**g**) at 40X magnification, stained with CD66b (green) and Hoechst (blue). **(h):** Bar graphs indicating the neutrophil infiltration in healthy controls (n = 6) and patients with PKDL (n = 12). Each horizontal bar represents the median (IQR). **(i):** Correlation of neutrophil infiltration (counts/field) with parasite load (no. of parasites/μg genomic DNA. **(j):** Bar graphs indicating the neutrophil infiltration in patients with macular (n = 6) and polymorphic PKDL (n = 6). Each horizontal bar represents the median (IQR).

The degree of dermal cell infiltration in macular (median 143; IQR, 106–185) and polymorphic PKDL (median 173; IQR, 142–195) was substantially higher than healthy controls (median 14.00; IQR, 12–16, Fig 1a and 1b). In the macular variant, the infiltrate was patchy, and presented as clusters in the upper dermis, whereas, in the polymorphic form, the infiltrate was dense and diffusely distributed throughout the dermis (Fig 1a), corroborating with previous studies [27]. The cellular infiltrate in the macular and polymorphic PKDL cases showed a significant correlation with parasite load, being r = 0.81, p<0.05 and r = 0.89, p<0.05 respectively.

### Status of lesional CD66b^+^ neutrophils in patients with PKDL

As mature human neutrophils express CD66b [31], neutrophils were identified in terms of CD66b positivity. In comparison to healthy controls, where CD66b^+^ neutrophils were absent, there was a substantial number present in PKDL cases (median, 58; IQR, 31–70 positive cells/FOV (Fig 1c–1h S1 Fig). The proportion of neutrophils relative to the total number of infiltrated cells at the lesional sites is approximately 9% (as determined at 10X magnification, following analysis of 5 fields). Notably, the other cell types that constitute the major infiltrate in PKDL were macrophages, CD8^+^ T cells and B cells, with a conspicuous absence of CD4^+^ T cells [27]. A direct quantitative comparison of neutrophil percentages with other major cell types was not attempted in this study, but a qualitative assessment suggests a hierarchical order of infiltration, macrophages > CD8^+^ T cells > B cells > neutrophils. This qualitative assessment is based on prior publications, which demonstrated the presence of CD8^+^ T cells and CD20^+^ B cells in PKDL [27], rather than relying solely on H&E staining. The proportion of CD66b^+^ neutrophils strongly correlated with the parasite burden (r = 0.90, p<0.01; Fig 1i and S1 Data), but moderately with disease duration (r = 0.42, p value non-significant), and lag period (r = 0.60, p value non-significant).

In both forms of PKDL, CD66b^+^ neutrophils showed a patchy distribution, mostly in the perivascular region (Fig 1 and S1 Data). A significant correlation between neutrophil infiltration and parasite load in macular and polymorphic PKDL was observed, being r = 0.80, p<0.05 and 0.91, p<0.05 respectively. On a lesional basis, macular and polymorphic PKDL demonstrated a comparable number of CD66b^+^ neutrophils (51; 10–72 and 62; 45–73 positive cells/FOV respectively, Fig 1f, 1g and 1j) and the differences between the two clinical forms was not statistically significant. Accordingly, the subsequent analysis was done considering PKDL as a single entity. The brightness and contrast settings were consistent across all figures (Fig 1c–1e); the staining in the images acquired from healthy controls (Fig 1c) appeared dimmer as compared to PKDL (Fig 1d and 1e) owing to minimal cell infiltration and corroborated with the IHC data (Fig 1a).

### Increased levels of neutrophil chemo-attractants in patients with PKDL

**Fig 2:**
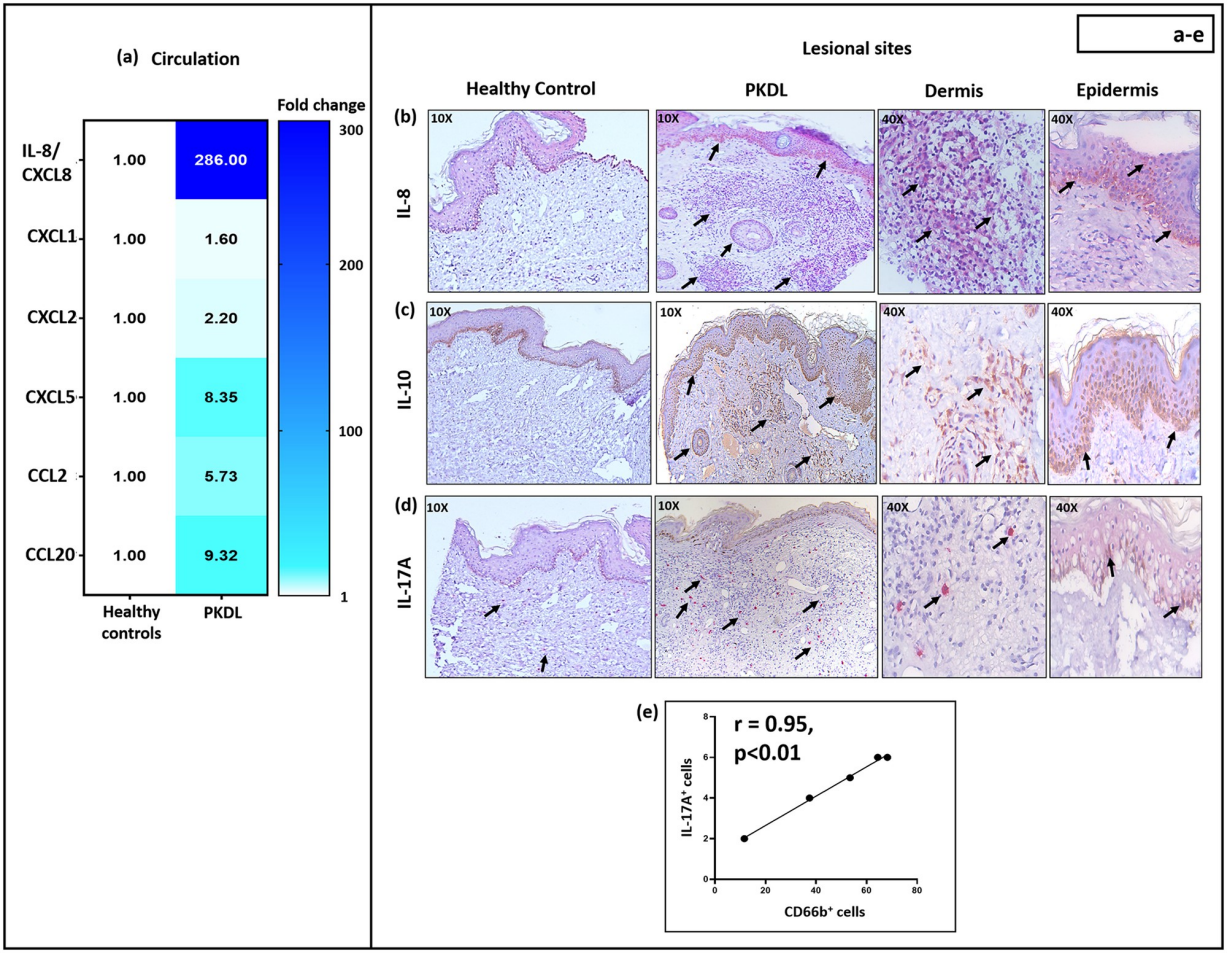
Levels of neutrophil chemo-attractants in patients with Post Kala-azar Dermal Leishmaniasis (PKDL). **(a):** Heatmap indicating the fold change increase in levels of circulatory neutrophil chemo-attractants in patients with PKDL (n = 20) with respect to healthy controls (n = 10). The colour bar represents the range of fold change (1–300), with dark blue representing highest fold change and white as baseline. **(b-d):** Representative immunohistochemical profiles showing the expression of IL-8 (**b**), IL-10 (**c**) and IL-17A (**d**) in skin biopsies of a healthy control (n = 5) and patient with PKDL (n = 5, 10X magnification). In PKDL, areas with positive staining are further imaged at 40X magnification. The chromogen colour red [IL-8 **(b)** and IL-17A **(d)**] or brown [IL-10 **(c)**] in the figures indicates the presence of positive stain (for IL-8, 10) or positively stained cells in IL-17A (indicated with black arrows). **(e)**: Correlation between the number of IL-17A^+^ cells and CD66b^+^ neutrophils at the lesional sites of patients with PKDL.

Cytokines and chemokines play a crucial role in initiating inflammatory responses by attracting neutrophils from the circulation to the tissues [32]. Given the perivascular distribution of neutrophils, plasma levels of neutrophil chemo-attractants IL-8/CXCL8, CXCL1, CXCL2, CXCL5, CCL2 and CCL20 were measured. In PKDL cases, all chemokines analysed were significantly upregulated *vis-à-vis* healthy controls, maximal enhancement being for IL-8/CXCL8; the number of CD66b^+^ neutrophils correlated positively with IL-8/CXCL8 and CXCL5 levels (Fig 2a Table 2 and S1 Data). Additionally, IL-8 was detectable in PKDL lesions (5/5), but not in healthy controls (5/5, Fig 2b); it was largely confined to the basal layer of the epidermis, with fewer IL-8^+^ cells present in the dermis. The expression of IL-8 was diffuse, and an individual cell count was not attempted.

**Table 2:** Circulatory levels of neutrophil chemo-attractants in Post Kala-azar Dermal Leishmaniasis (PKDL).

Cytokines/Chemokines	PKDL (pg/ml), [n = 20]	Healthy controls (pg/ml), [n = 10]	p value	Correlation (r), with lesional CD66b^+^ cells/field
IL-8/CXCL8	7412 (1604–36638)	25.91 (16.96–75.32)	p<0.0001	r = 0.54, p<0.05
CXCL1	425.90 (339.20–926.30)	266.18 (186.40–382.90)	p<0.01	r = -0.58 (ns)
CXCL2	101.50 (85.18–115.30)	46.13 (39.86–79.21)	p<0.01	r = 0.36 (ns)
CXCL5	5204 (2615–7232)	623.23 (457.60–1022)	p<0.0001	r = 0.97, p<0.001
CCL2	321.90 (68.99–936.70)	56.17 (46.91–66.97)	p<0.01	r = 0.35 (ns)
CCL20	20.43 (14.39–37.73)	2.19 (1.68–5.52)	p<0.0001	r = 0.60 (ns)

The circulatory levels of neutrophil chemo-attractants were measured as described in Materials and methods. Values are stated in median (IQR). ns: not significant.

### Status of inflammatory milieu in patients with PKDL

The plasma cytokine profiles of PKDL cases indicated a mixed pro- and anti-inflammatory response, with elevated levels of Th_1_ (IL-6 and IFN-γ), Th_2_ (IL-4) and T_reg_ (IL-10), along with a conspicuous absence of Th_17_ (IL-17, IL-22 and 23) cytokines (Table 3 and S1 Data). The presence of CD66b^+^ neutrophils in lesions correlated strongly with circulating levels of IL-10 (Table 3 and S1 Data); additionally, IL-10 was diffusely expressed in the dermis and epidermis of PKDL patients (5/5), but not in healthy controls (5/5, Fig 2c), accordingly, an individual cell count was not attempted. Although not detectable in plasma, IL-17A was detected in PKDL lesions (Fig 2d), with epidermal expression in 2/5 patients, and dermal expression in 5/5 cases. In the dermis of PKDL cases, the number of IL-17A^+^ cells were quantified, (median 5 cells/FOV; IQR, 3–7 vs. 0.6; 0–1 in healthy controls), and significantly correlated with neutrophilic infiltration (r = 0.95, p<0.01, Fig 2e). With regard to the lesional infiltration of CD66b^+^ neutrophils (Fig 1d–1h), it was predominantly localized in the perivascular regions. The IL-17A^+^ cells were dispersed throughout the dermis and in close proximity to the perivascular areas (Fig 2d).

**Table 3:** Status of the circulatory inflammatory milieu in patients with Post Kala-azar Dermal Leishmaniasis (PKDL).

Cytokines	PKDL (pg/ml) [n = 20]	Healthy controls (pg/ml) [n = 10]	p value	Correlation (r) with lesional CD66b^+^ cells/field
IL-6	82.80 (20.64–167.7)	7.71 (2.06–38.10)	p<0.001	r = -0.77 (ns)
IFN-γ	71.63 (38.56–147.50)	27.42 (21.94–30.39)	p<0.01	r = -0.79 (ns)
IL-4	61.25 (52.31–77.35)	41.27 (24.97–50.40)	p<0.01	r = -0.25 (ns)
IL-10	143.50 (122.70–160.80)	7.35 (5.69–13.34)	p<0.0001	r = 0.60, p<0.05
IL-17	1.23 (1.04–1.86)	1.07 (0.94–1.31)	ns	r = -0.56 (ns)
IL-23	24.90 (17.39–35.56)	20.15 (3.28–28.83)	ns	r = 0.05 (ns)
IL-22	4.41 (3.12–5.74)	3.43 (3.12–4.08)	ns	r = 0.03 (ns)

The levels of cytokines were measured and their correlation with CD66b^+^ neutrophils analysed as described in Materials and methods. ns: not significant. Values are stated in median (IQR).

### Status of neutrophil activation in patients with PKDL

**Fig 3:**
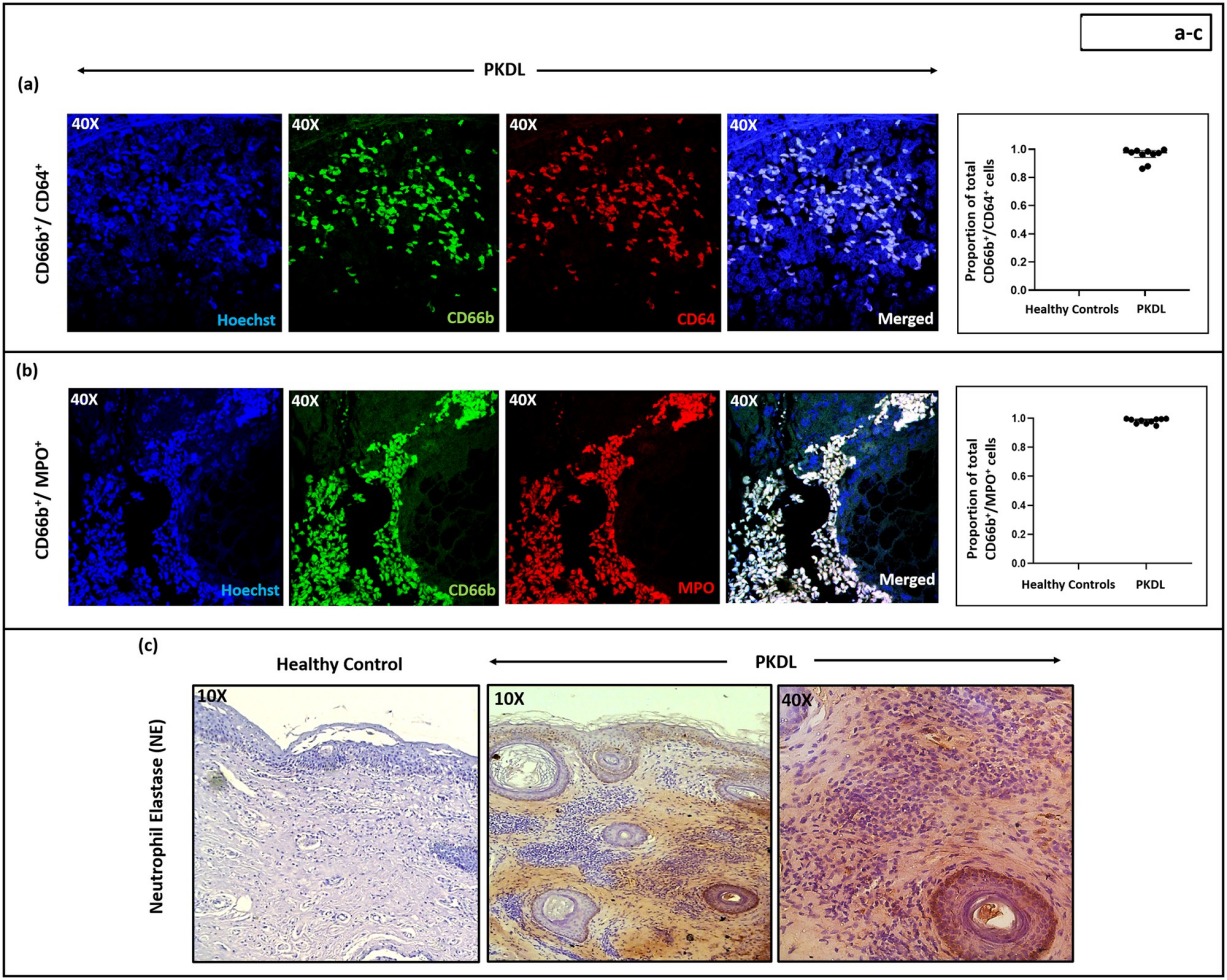
Activation status of CD66b^+^ neutrophils at the lesional sites of patients with Post Kala-azar Dermal Leishmaniasis (PKDL). **(a-b):** Representative immunofluorescence profiles showing the expression of **(a)** CD64 [n = 10, red] and (**b)** Myeloperoxidase (MPO) [n = 10, red], along with CD66b [green] positive cells in dermal biopsies of patients with PKDL (magnification, 40X). Co-localization of CD66b with CD64^+^ or MPO^+^ cells are indicated as ‘merged’ and counterstained with Hoechst [blue]. Scatter plots showing the proportion of double positive cells [(**a)** CD66b^+^/CD64^+^ and (**b)** CD66b^+^/MPO^+^]; wherein each dot (●) represents a patient. Error bars represent the median (IQR). **(c):** Representative immunohistochemical profiles showing the expression of neutrophil elastase (NE) in dermal biopsies of a healthy control (n = 6) and patient with PKDL (n = 8) at magnification 10X. In PKDL, areas with positive staining are further imaged at 40X magnification.

As activated neutrophils express CD64 [F_c_γR, 33,34], its expression in PKDL lesions was assessed. Patients with PKDL demonstrated raised numbers of CD66b^+^CD64^+^ cells (median, 56; IQR, 31–66 cells/FOV), 100% of infiltrated neutrophils were activated, whereas in healthy controls, the neutrophils were not activated (Fig 3a and S2 Fig). Interestingly, these activated neutrophils at the lesional sites demonstrated a significant correlation with disease duration, being r = 0.63, p<0.05.

Myeloperoxidase (MPO), an azurophilic granule marker of neutrophils is associated with activation and degranulation [35] and was abundantly present in lesional CD66b^+^ neutrophils (median, 57; IQR 30–69 cells/FOV, Fig 3b and S2 Fig), all the infiltrated neutrophils were activated and showed a positive correlation with CD66b^+^ CD64^+^ cells (r = 0.85, p<0.05). Neutrophil elastase (NE) is a serine protease expressed in neutrophil primary granules and is involved with neutrophil extracellular traps [NETs, 36]; in 7/12 PKDL cases, there was lesional expression of NE (Fig 3c), but not in healthy controls.

### Status of MMP9 in patients with PKDL

**Fig 4:**
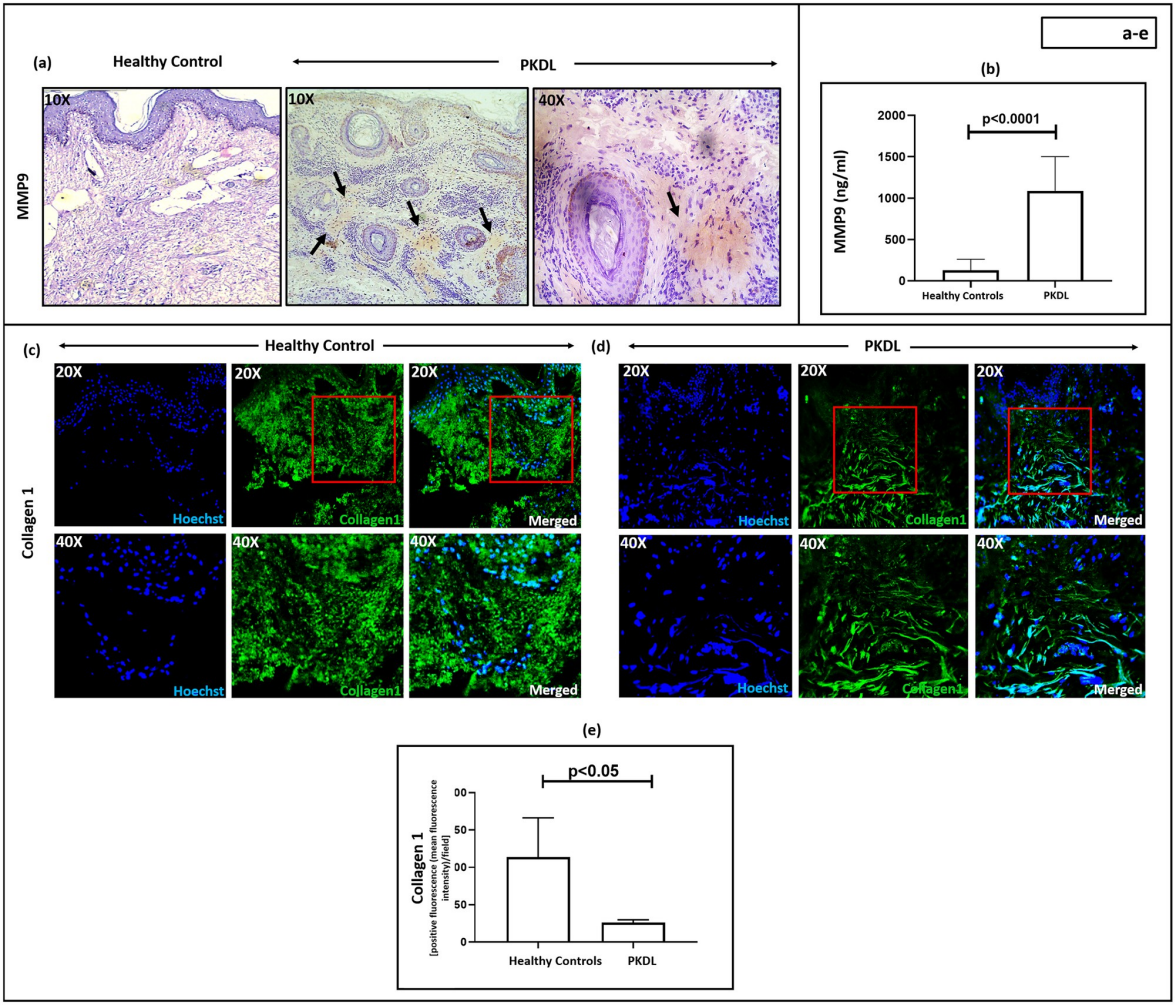
Status of MMP9 and Collagen I in Post Kala-azar Dermal Leishmaniasis (PKDL). **(a):** Representative immunohistochemical profiles showing the expression of matrix metalloproteinase 9 (MMP9) in dermal biopsies of a healthy control (n = 6) and patient with PKDL (n = 11) at 10X magnification. Presence of MMP9 has been indicated with black arrowheads. In PKDL, areas with positive staining are further imaged at 40X magnification. **(b):** Bar graphs indicating the levels of circulatory MMP9 (ng/ml) in healthy controls (n = 10) and patients with PKDL (n = 20). Each horizontal bar represents the median (IQR). **(c-e):** Representative immunofluorescence profiles showing the expression of collagen I in dermal biopsies of a healthy control (**c**) and patient with PKDL (**d**), at 20X and 40X magnification. The red squares in 20X images represent the area that was imaged at 40X magnification. Bar graphs **(e)** showing the expression of collagen I in healthy controls (n = 7) and patients with PKDL (n = 7). Each horizontal bar represents the median (IQR).

Neutrophils harbour MMP9 in their azurophilic granules and can be activated by NE [37], and this release of MMP9 augments inflammatory responses [38]. In comparison to healthy controls where MMP9 was absent in the skin, its expression in PKDL lesions was moderate (Fig 4a and S1 Data). Additionally, plasma levels of MMP9 were 8.65-fold higher than healthy controls, 1117.65 (693.33–1514.18) vs. 129.15 (71.18–260.53) ng/ml respectively, p<0.0001 (Fig 4b and S1 Data).

### Collagen I expression in patients with PKDL

As MMP9 can modulate the skin architecture, we examined expression of collagen I, an extracellular matrix protein that provides the skin a structural framework. The expression of collagen I in PKDL cases was significantly decreased [median 26; IQR, 20–29, positive fluorescence (mean fluorescence intensity)/field, p<0.01] as compared to healthy controls (median, 183; IQR, 111–226, positive fluorescence (mean fluorescence intensity) /field, Fig 4c–4e). The reduced fluorescence intensity observed in PKDL, is attributed to the decreased presence of collagen I in the lesional tissue (Fig 4c–4e).

## Discussion

Dermal leishmaniasis includes a diverse spectrum of clinical and immunological manifestations [39], as LCL exhibits a Th_1_ dominance, whereas MCL a Th_2_ dominance [40]. PKDL is midway, as it demonstrates a mixed Th_1_-Th_2_ profile [40,41]. PKDL, unlike other dermal forms, has its origins in the systemic disease, VL [42]. The immunopathogenesis of dermal leishmaniasis involves recruitment of neutrophils during the acute, active and ulcerative phases of lesion development [39]. In LCL, neutrophils generally infiltrate in proximity with keratinocytes and are proposed to mediate tissue destruction and resultant ulceration [43]. Similarly, neutrophil abundance has been reported in MCL which present with ulcerated or necrotized lesions [18]. In non-ulcerative DCL, there is uncontrolled parasite dissemination, with neutrophils migrating to the dermis [39]. PKDL differs from these dermal leishmaniases in that there is hypopigmentation in over 50% of cases, and in all cases, ulceration is conspicuously absent even when the disease duration is prolonged [2]. To date, there is no animal model for PKDL and knowledge regarding the presence, distribution and role of neutrophils remains to be delineated.

At the lesional sites of PKDL cases, irrespective of the form, there was an increased infiltration of immune cells (Fig 1a), and a deeper investigation established the presence of CD66b^+^ neutrophils (Fig 1c–1j). However, the status of neutrophils in PKDL has been largely overshadowed by the focus on macrophages, dendritic cells and T cells [27,42,44]. This may be attributed to PKDL cases having a conspicuous absence of any severe tissue damage e.g., ulceration, where neutrophils are consistently proposed as the major proponent [39]. The dermal CD66b^+^ neutrophils showed a patchy distribution mainly in the perivascular regions (Fig 1d–1g and S1 Fig) in PKDL. Neutrophils, being terminally differentiated cells, their presence in tissue typically indicates homing and recruitment. In this light, previous studies of PKDL lesions have failed to identify Ki67^+^ cells, confirming absence of cellular proliferation at lesional sites [27] and substantiated their dermal homing. This assertion was strengthened by an increased presence of neutrophil-attracting chemokines in plasma and tissue of PKDL patients (Fig 2). Furthermore, a positive correlation between the number of amastigotes in the lesions and neutrophil numbers was observed (Fig 1i), though whether this is the cause or effect cannot be determined.

Circulating chemo-attractants that induce recruitment of neutrophils to sites of tissue injury include IL-8/CXCL8 [45], CXCL1, CXCL2 [46], CXCL5 [47], CCL2 [48] and CCL20 [49]. In CL, increased levels of CXCL8 and CXCL5 mediated recruitment of neutrophils and amplified disease pathogenesis [50]. Although, neutrophil recruitment has been observed in MCL, LCL or DCL [39], an association, if any, with IL-8 or CXCL5 has not been studied. In patients with PKDL, we observed raised circulating (Fig 2a and Table 2) and lesional expression of IL-8 (Fig 2b), akin to the scenario in leprosy, another intra-macrophagic dermal disorder [51]. Furthermore, neutrophils isolated from patients with lepromatous leprosy, with or without erythema nodosum leprosum (ENL), released IL-8 upon *ex-vivo* stimulation with *M*. *leprae* [52]. Keratinocytes, a rich source of IL-8, have been demonstrated to exert a regulatory influence on *ex-vivo Leishmania* infections, and induce influx of neutrophils [53]. Likewise, in PKDL the increased presence of epidermal IL-8 strongly endorsed the contributory role of keratinocytes in neutrophil recruitment (Fig 2b).

During *Streptococcus pneumoniae* infection, infiltration of neutrophils into the lungs serve as a cellular source of IL-10 and mediate an effective Th2-mediated response immune response necessary for host survival [54]. In PKDL, levels of IL-10 were raised in circulation, and at lesional sites, as also strongly correlated with the proportion of lesional neutrophils (Table 3 and Fig 2c); however, the cellular source of lesional IL-10 was not delineated. Furthermore, the role of IL-10 in modulating inflammation at the lesional sites in PKDL has been associated with its expression in CD3^+^CD8^+^ T cells, increased parasite burden and high antibody levels, including IgG1 and IgG3 [42].

The spectrum of T cell responses varies widely in different forms of human CL, as in MCL and LCL, there is a Th_1_ predominance associated with extensive tissue damage, while anergic DCL reflects a Th2 dominance [ADCL, 40]. PKDL appears positioned in the middle of the spectrum as it exhibited an upregulation of Th_1_ and Th_2_ cytokines, coupled with a strong T_reg_ response [IL-10, 54]. In addition, the absence of a Th_17_ response in circulation of PKDL cases in terms of IL-17 and IL-23 (Table 3) indicated a discordance with previous studies [55]. However, the correlation between dermal IL-17A expression and neutrophil abundance (Fig 2d and 2e) suggested that IL-17 may facilitate neutrophil recruitment, as reported in MCL [18]. Additionally, other cells, such as skin-resident leukocytes (mast cells) are important sources of IL-17 in type 3 inflammation that also correlates with neutrophilic infiltration [56]. In summation, a substantial body of evidence endorses a positive correlation between T cells and neutrophils [56], and this could well be a bi-directional phenomenon, as neutrophils can attract T cells [57]. Accordingly, one may propose that these infiltrated neutrophils possibly augment the dermal homing of CD8^+^ T cells in PKDL [58]. Furthermore, in *Leishmania* infected mice, the transcriptomic profile under hypoxic conditions correlated with an enhanced presence of neutrophils that in turn stimulated the production of granzyme B by lesional CD8^+^ T cells [59].

In skin autoinflammation, IL-26 (primarily produced by Th_17_ cells), is proposed to have a role in activation of neutrophils. Although this was not assessed in this study, the status of IL-22, which belongs to the same IL-20 cytokine subfamily with shared receptor subunits and similar functions [60] was evaluated, wherein no significant differences was demonstrated in the circulating levels of PKDL patients vs. healthy controls (Table 3), suggestive of a minimal role for Th_17_ in PKDL.

In PKDL, most neutrophils at the lesional sites were functionally active, as judged by the expression of CD64, MPO and NE (Fig 3a, 3b and 3c). In leprosy, the upregulation of CD64 is associated with progression of inflammation [61]. Similarly, in PKDL a considerable proportion of the infiltrated neutrophils demonstrated CD64 positivity (Fig 3a). The presence of MPO, an azurophilic granule marker, indicates neutrophil activation, as demonstrated in leprosy [61], and formation of neutrophil extracellular traps in various models of inflammation [62]. Accordingly, the raised presence of MPO and their strong correlation with CD64^+^ neutrophils, endorsed the activated status of neutrophils (Fig 3b). Neutrophil elastase (NE), a marker for presence of neutrophil extracellular traps (NETs), that trap and kill pathogens following the release of antimicrobial compounds [i.e., NE, cathepsin G, histones etc., 63] was also enhanced in PKDL (Fig 3c), and substantiated the potential host-protective role of neutrophils; however, as amastigotes are resistant to NETosis, disease progression continued unabated. Interestingly, unlike the other forms of CL, the activated neutrophils did not translate into development of ulceration, and was further supported by the absence of any granuloma formation [3]. Furthermore, there was a conspicuous absence of neutrophils in the epidermis, varying degrees of basal cell degeneration [64] along with a strong presence of IL-10. This fuels the necessity of deeper understanding of the disease, in that PKDL is perhaps not just a ‘wound’, rather a complex network of immune cells and inflammatory milieu culminating in a distinct dermal pathology.

Irrespective of the trigger being infectious or non-infectious in origin, neutrophils are a predominant source of MMP9, a proteolytic enzyme majorly involved in inflammation and tissue remodelling via collagen degradation. In patients with leprosy presenting with inflammatory reversal reactions and erythema nodosum leprosum (ENL), the levels of MMP9 in tissue and circulation were elevated [65]. Similarly, in human CL or MCL, the raised levels of MMP9 were associated with ulceration [66] and in PKDL, raised levels have been reported [67], (Fig 4a and 4b); importantly, it was not accompanied by ulceration. In PKDL, as compared to LCL and MCL [66], the levels of MMP9 were relatively lower (Fig 4a), which could account for the lower proportion of tissue damage.

MMP9 not only causes degradation of the matrix components, but also facilitates transmigration of host immune defence cells, including macrophages and lymphocytes and thus augments disease progression [42]. Collagen, an intrinsic component of the ECM of human skin can be degraded by MMPs [68]. With decreased expression of collagen I (Fig 4c–4e) and concomitant increased lesional expression of MMP9 (Fig 4a), it is tempting to speculate that MMP9 via disruption of matrix integrity caused loosening of the matrix component that facilitated entry of immune cells, necessitating a deeper analysis of other ECM components. Taken together, it can be stated that activated neutrophils in PKDL cases supported disease progression by contributing towards a weakened matrix integrity, which facilitated the influx of immune cells, e.g., macrophages, T and B lymphocytes. In addition, it should be noted that neutrophil infiltration and its associated functionalities as demonstrated in the study are not unique to PKDL, as similar patterns have been observed in other diseases such as leprosy, CL, DCL, LCL, MCL etc. [18,39,43,52,61].

Studies in PKDL have demonstrated that an IFN-γ associated pro-inflammatory landscape resulted in melanocyte loss/destruction with impairment of the melanogenesis pathway (reduction in key melanogenesis enzymes, *MITF*, *TYR* and *TYRP1*), and possibly accounted for the hypopigmentation [28]. In vitiligo, a chronic hypomelanotic disorder, an increased presence of MMP9 is implicated with loss of melanocytes and associated hypopigmentation, via decreased expression of E-cadherin at the peri-lesional sites [69]. As E-cadherin is critical for attachment of keratinocytes with melanocytes, a decrease in E-cadherin could account for the loss of melanocytes/melanocyte death, resulting in hypopigmentation [28]. Indeed, in PKDL, a decreased expression of E-cadherin has been demonstrated (Mitali Chatterjee, personal communication), and coupled with the enhanced presence of MMP9 (Fig 4a), it can be proposed that in PKDL, MMP9 plays a role in the pathogenesis of hypopigmentation. Taken together, in PKDL, a chronic dermatosis, neutrophils as a component of the chronic immune response play a multifaceted role. The activation of neutrophils at the lesional sites translates into the release of neutrophil elastase and MMP9 (Figs 3 and 4), that causes a decrease in the expression of collagen I, an important matrix component. This reduction of collagen 1 at the lesional site can potentially facilitate the influx of other immune cells, such as macrophages, which being the preferred host cell for parasite replication and survival, can pave the way for disease progression.

### Limitations of the study

A few notable limitations inherent to the study include non-inclusion of skin biopsies from unaffected areas, as patients were generally willing to provide a maximum of two biopsies, and were collected exclusively from active lesional sites. Secondly, due to the limited availability of biological material, not all markers could be evaluated in each patient, necessitating a random selection of samples for the determination of individual parameters, wherein a minimum of 5–6 samples were analysed per assay. Third, the relatively small sample size of the study may impact on the rigor of the analysis and limit the robustness of the analyses; however, the study aimed to have a distribution of macular and polymorphic PKDL cases (1:1), consistent with the current epidemiological status of PKDL [2]. Lastly, healed VL cases as controls would perhaps have been a better control arm *vis-a-vis* employment of uninfected, *Leishmania*-negative samples, as they do not comprehensively elucidate neutrophil-related features that are specific for PKDL. However, obtaining tissue biopsy specimens from a healed VL patient with no antecedent symptoms of papules, nodules, or lesions, is challenging as it is an invasive procedure.

## Supporting information

S1 Fig Representative profile of H&E stained section showing the presence of neutrophils (indicated with black arrows) from a dermal biopsy of a PKDL patient (100X magnification). (TIF)

S2 Fig Status of CD64 and MPO in CD66b^+^ neutrophils from healthy controls. Representative immunofluorescence profiles showing the expression of CD64 [red] and MPO [red] along with CD66b [green] cells in biopsies of healthy controls (n = 6, magnification, 40X). Co-localization of CD66b with CD64^+^ or MPO^+^ cells are indicated as ‘merged’ and counterstained with Hoechst [blue].
(TIF)

S1 Data The minimal data set includes all raw data utilized in the statistical analyses presented in the figures, ensuring transparency and reproducibility of the results. (XLSX)
