# Supplementary figures and images for: Role of neutrophils in the pathogenesis of Post Kala-azar Dermal Leishmaniasis (PKDL)
Source: PLoS Negl Trop Dis. 2024 Nov 27;18(11):e0012655. doi: 10.1371/journal.pntd.0012655 (PMC11602034; doi:10.1371/journal.pntd.0012655)

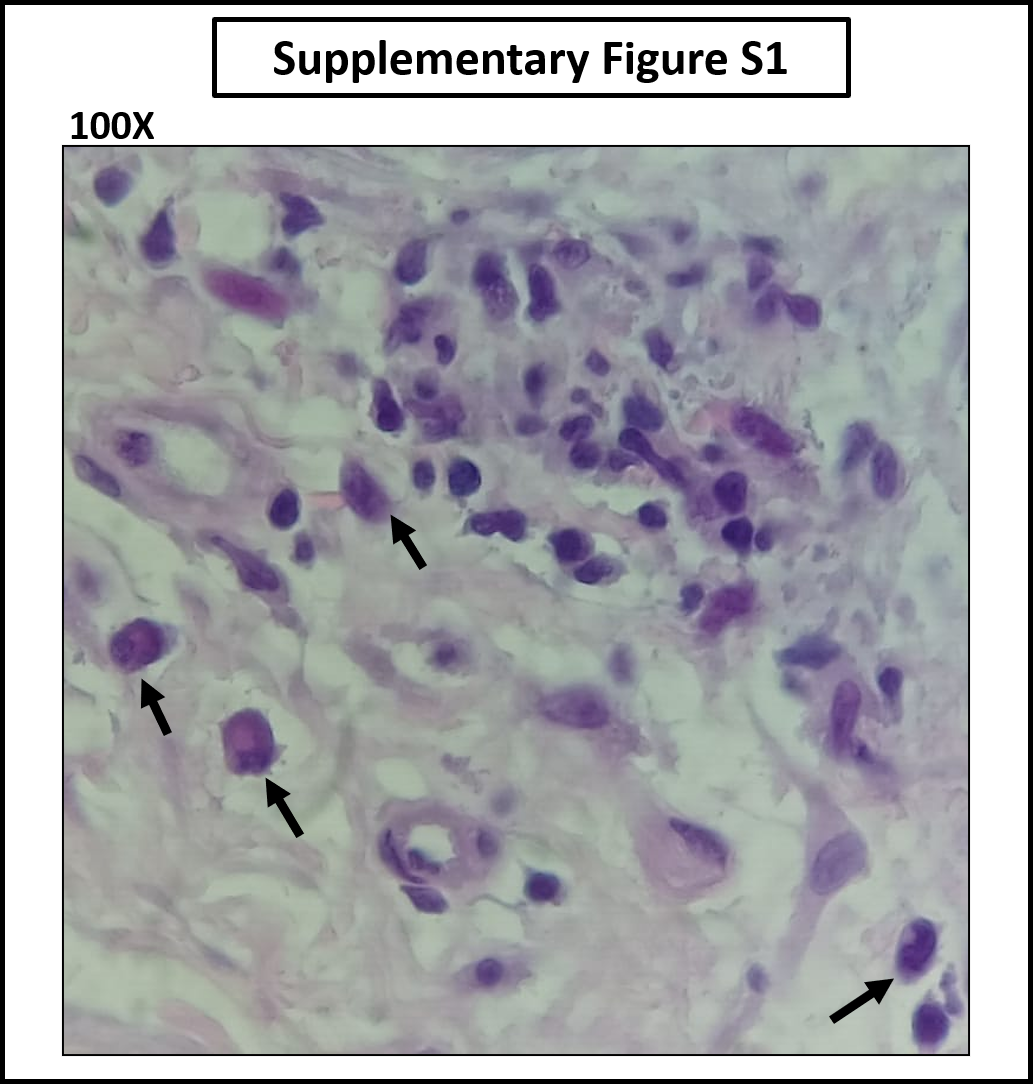

Supplement: S1 Fig — (TIF) [file pntd.0012655.s001.tif]

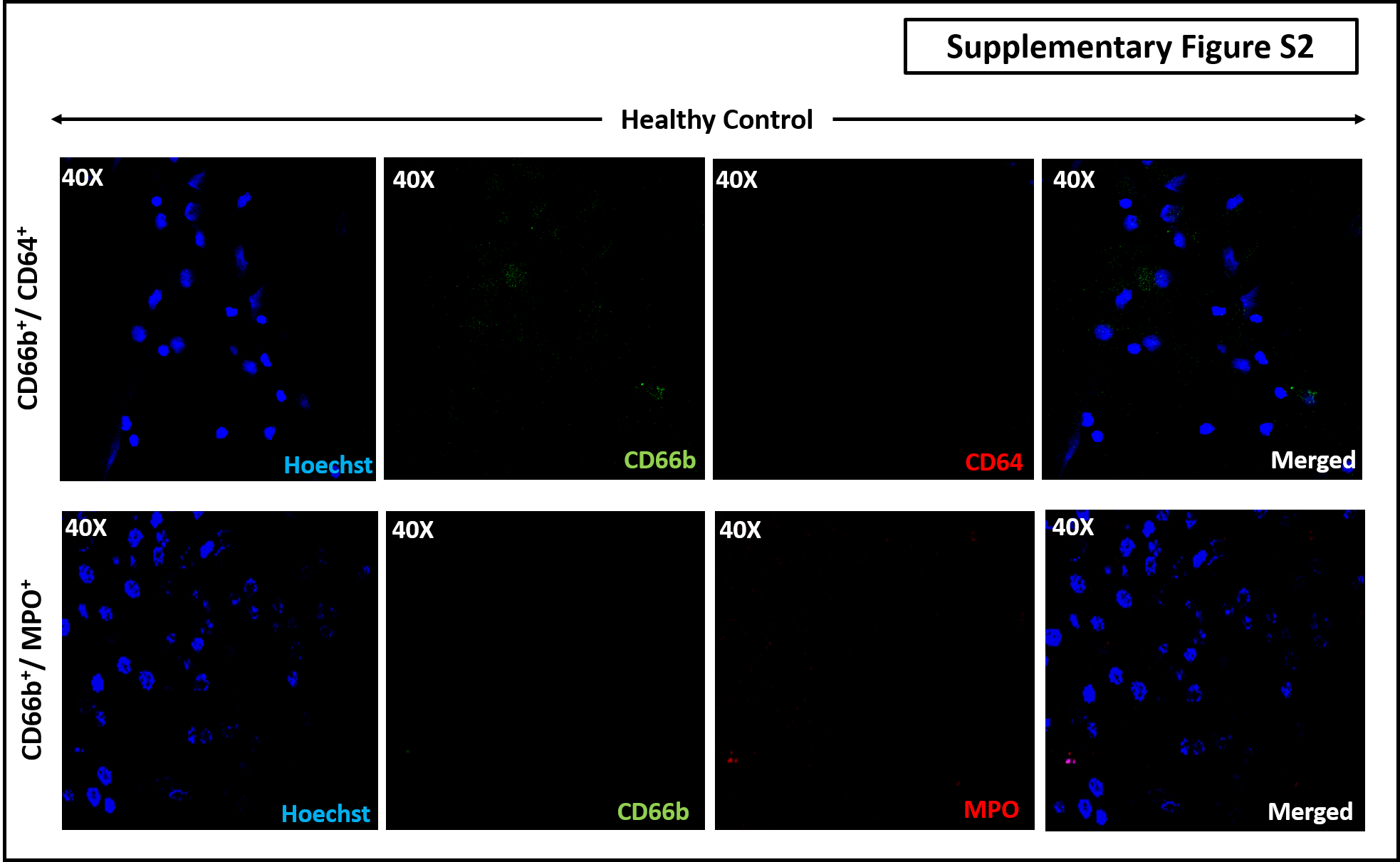

Supplement: S2 Fig — Representative immunofluorescence profiles showing the expression of CD64 [red] and MPO [red] along with CD66b [green] cells in biopsies of healthy controls (n = 6, magnification, 40X). Co-localization of CD66b with CD64+ or MPO+ cells are indicated as ‘merged’ and counterstained with Hoechst [blue]. (TIF) [file pntd.0012655.s002.tif]
